# Inheritance of deleterious mutations at both *BRCA1* and *BRCA2* in an international sample of 32,295 women

**DOI:** 10.1186/s13058-016-0768-3

**Published:** 2016-11-11

**Authors:** Timothy R. Rebbeck, Tara M. Friebel, Nandita Mitra, Fei Wan, Stephanie Chen, Irene L. Andrulis, Paraskevi Apostolou, Norbert Arnold, Banu K. Arun, Daniel Barrowdale, Javier Benitez, Raanan Berger, Pascaline Berthet, Ake Borg, Saundra S. Buys, Trinidad Caldes, Jonathan Carter, Jocelyne Chiquette, Kathleen B. M. Claes, Fergus J. Couch, Cezary Cybulski, Mary B. Daly, Miguel de la Hoya, Orland Diez, Susan M. Domchek, Katherine L. Nathanson, Katarzyna Durda, Steve Ellis, EMBRACE, D. Gareth Evans, Lenka Foretova, Eitan Friedman, Debra Frost, Patricia A. Ganz, Judy Garber, Gord Glendon, Andrew K. Godwin, Mark H. Greene, Jacek Gronwald, Eric Hahnen, Emily Hallberg, Ute Hamann, Thomas V. O. Hansen, HEBON, Evgeny N. Imyanitov, Claudine Isaacs, Anna Jakubowska, Ramunas Janavicius, Katarzyna Jaworska-Bieniek, Esther M. John, Beth Y. Karlan, Bella Kaufman, KConFab investigators, Ava Kwong, Yael Laitman, Christine Lasset, Conxi Lazaro, Jenny Lester, Niklas Loman, Jan Lubinski, Siranoush Manoukian, Gillian Mitchell, Marco Montagna, Susan L. Neuhausen, Heli Nevanlinna, Dieter Niederacher, Robert L. Nussbaum, Kenneth Offit, Edith Olah, Olufunmilayo I. Olopade, Sue Kyung Park, Marion Piedmonte, Paolo Radice, Christine Rappaport-Fuerhauser, Matti A. Rookus, Caroline Seynaeve, Jacques Simard, Christian F. Singer, Penny Soucy, Melissa Southey, Dominique Stoppa-Lyonnet, Grzegorz Sukiennicki, Csilla I. Szabo, Mariella Tancredi, Manuel R. Teixeira, Soo-Hwang Teo, Mary Beth Terry, Mads Thomassen, Laima Tihomirova, Marc Tischkowitz, Amanda Ewart Toland, Aleksandra Toloczko-Grabarek, Nadine Tung, Elizabeth J. van Rensburg, Danylo Villano, Shan Wang-Gohrke, Barbara Wappenschmidt, Jeffrey N. Weitzel, Jamal Zidan, Kristin K. Zorn, Lesley McGuffog, Douglas Easton, Georgia Chenevix-Trench, Antonis C. Antoniou, Susan J. Ramus

**Affiliations:** 1 Department Epidemiology, Dana Farber Cancer Institute and Harvard T.H. Chan School of Public Health, 1101 Dana Building, 450 Brookline Avenue, Boston, MA USA; 2 Department of Biostatistics and Epidemiology, Perelman School of Medicine at the University of Pennsylvania, Philadelphia, PA USA; 3 Biostatistics Unit, Group Health Research Institute, Seattle, WA USA; 4 Department of Preventive Medicine, Keck School of Medicine, USC/Norris Comprehensive Cancer Center, University of Southern California, California, USA; 5 Department of Genetics and Computational Biology, QIMR Berghofer Institute of Medical Research, Brisbane, Australia; 6 Centre for Cancer Genetic Epidemiology, Department of Public Health and Primary Care, University of Cambridge, Cambridge, UK; 7 Lunenfeld-Tanenbaum Research Institute, Mount Sinai Hospital, Toronto, Ontario M5G 1X5 Canada; 8 Departments of Molecular Genetics and Laboratory Medicine and Pathobiology, University of Toronto, Toronto, Ontario Canada; 9 Molecular Diagnostics Laboratory, (INRASTES) Institute of Nuclear and Radiological Sciences and Technology, National Centre for Scientific Research “Demokritos”, Patriarchou Gregoriou & Neapoleos str. Aghia Paraskevi Attikis, Athens, Greece; 10 Department of Gynaecology and Obstetrics, University Hospital of Schleswig-Holstein, Campus Kiel, Christian-Albrechts University, Kiel, Germany; 11 Department of Breast Medical Oncology and Clinical Cancer Genetics Program, University Of Texas MD Anderson Cancer Center, 1515 Pressler Street, CBP 5, Houston, TX USA; 12 Human Genetics Group, Spanish National Cancer Centre (CNIO), Madrid, Spain; 13 Biomedical Network on Rare Diseases (CIBERER), Madrid, Spain; 14 Human Genotyping (CEGEN) Unit, Human Cancer Genetics Program, Spanish National Cancer Research Centre (CNIO), Madrid, Spain; 15 The Institute of Oncology, Chaim Sheba Medical Center, Ramat Gan, 52621 Israel; 16 Centre François Baclesse, 3 avenue Général Harris, Caen, France; 17 Department of Oncology, Clinical Sciences, Lund University and Skåne University Hospital, Lund, Sweden; 18 Department of Medicine, Huntsman Cancer Institute, 2000 Circle of Hope, Salt Lake City, UT 84112 USA; 19 Molecular Oncology Laboratory, Hospital Clinico San Carlos, IdISSC (El Instituto de Investigación Sanitaria del Hospital Clínico San Carlos), Martin Lagos s/n, Madrid, Spain; 20 Gynaecological Oncology, The University of Sydney Cancer Centre, Royal Prince Alfred Hospital, Sydney, Australia; 21 Unité de recherche en santé des populations, Centre des maladies du sein Deschênes-Fabia, Hôpital du Saint-Sacrement, 1050 chemin Sainte-Foy, Québec Canada; 22 Center for Medical Genetics, Ghent University, De Pintelaan 185, 9000 Gent, Belgium; 23 Department of Laboratory Medicine and Pathology, and Health Sciences Research, Mayo Clinic, 200 First Street SW, Rochester, Minnesota USA; 24 Department of Genetics and Pathology, Pomeranian Medical University, Polabska 4, Szczecin, Poland; 25 Division of Population Science, Fox Chase Cancer Center, 333 Cottman Avenue, Philadelphia, PA 19111 USA; 26 Oncogenetics Group, Vall d’Hebron Institute of Oncology (VHIO), Clinical and Molecular Genetics Area, Vall d’Hebron University Hospital, Passeig Vall d’Hebron 119-129, Barcelona, Spain; 27 Department of Medicine, Abramson Cancer Center, Perelman School of Medicine at the University of Pennsylvania, Philadelphia, PA USA; 28 Centre for Cancer Genetic Epidemiology, Department of Public Health and Primary Care, University of Cambridge, Strangeways Research Laboratory, Worts Causeway, Cambridge, UK; 29 Genomic Medicine, Manchester Academic Health Sciences Centre, Institute of Human Development, Manchester University, Central Manchester University Hospitals NHS Foundation Trust, Manchester, UK; 30 Department of Cancer Epidemiology and Genetics, Masaryk Memorial Cancer Institute, Zluty kopec 7, Brno, 65653 Czech Republic; 31 The Susanne Levy Gertner Oncogenetics Unit, Institute of Human Genetics, Chaim Sheba Medical Center, Ramat Gan, 52621 Israel; 32 Sackler Faculty of Medicine, Tel Aviv University, Ramat Aviv, 69978 Israel; 33 UCLA Schools of Medicine and Public Health, Division of Cancer Prevention & Control Research Jonsson Comprehensive Cancer Center, 650 Charles Young Drive South, Room A2-125 HS, Los Angeles, CA 90095-6900 USA; 34 Ontario Cancer Genetics Network: Samuel Lunenfeld Research Institute, Mount Sinai Hospital, Toronto, Ontario M5G 1X5 Canada; 35 Department of Pathology and Laboratory Medicine, 3901 Rainbow Boulevard, 4019 Wahl Hall East, MS 3040 Kansas, USA; 36 University of Kansas Medical Center, Kansas City, Kansas USA; 37 Clinical Genetics Branch, DCEG, NCI, NIH, 9609 Medical Center Drive, Room 6E-454, Bethesda, MD USA; 38 Center for Hereditary Breast and Ovarian Cancer, Center for Integrated Oncology (CIO) and Center for Molecular Medicine Cologne (CMMC), Medical Faculty, University of Cologne and University Hospital Cologne, Cologne, Germany; 39 Department of Health Sciences Research, Mayo Clinic, 13400 E. Scottsdale Blvd., Scottsdale, AZ USA; 40 Molecular Genetics of Breast Cancer, German Cancer Research Center (DKFZ), Im Neuenheimer Feld 580, 69120 Heidelberg, Germany; 41 Center for Genomic Medicine, Rigshospitalet, Copenhagen University Hospital, Blegdamsvej 9, DK-2100 Copenhagen, Denmark; 42 The Hereditary Breast and Ovarian Cancer Research Group Netherlands (HEBON) Coordinating center: Netherlands Cancer Institute, Amsterdam, The Netherlands; 43 N.N. Petrov Institute of Oncology, St.-Petersburg, 197758 Russia; 44 Lombardi Comprehensive Cancer Center, Georgetown University, 3800 Reservoir Road NW, Washington, DC USA; 45 Department of Molecular and Regenerative Medicine, Vilnius University Hospital Santariskiu Clinics, Hematology, oncology and transfusion medicine center, Santariskiu st, Vilnius, Lithuania; 46 State Research Institute Centre for Innovative medicine, Zygymantu st. 9, Vilnius, Lithuania; 47 Department of Epidemiology, Cancer Prevention Institute of California, 2201 Walnut Avenue, Suite 300, Fremont, CA 94538 USA; 48 Women’s Cancer Program at the Samuel Oschin Comprehensive Cancer Institute, Cedars-Sinai Medical Center, 8700 Beverly Boulevard, Suite 290W, Los Angeles, CA USA; 49 Kathleen Cuningham Consortium for Research into Familial Breast Cancer, Peter MacCallum Cancer Center, Melbourne, Australia; 50 The Hong Kong Hereditary Breast Cancer Family Registry; Cancer Genetics Center, Hong Kong Sanatorium and Hospital, Hong Kong, Hong Kong; 51 Department of Surgery, The University of Hong Kong, Hong Kong, Hong Kong; 52 Unité de Prévention et d’Epidémiologie Génétique, Centre Léon Bérard, 28 rue Laënnec, Lyon, France; 53 Molecular Diagnostic Unit, Hereditary Cancer Program, IDIBELL (Bellvitge Biomedical Research Institute) Catalan Institute of Oncology, Gran Via de l’Hospitalet, 199-203, 08908, L’Hospitalet Barcelona, Barcelona, Spain; 54 Department of Oncology, Lund University Hospital, Lund, Sweden; 55 Unit of Medical Genetics, Department of Preventive and Predictive Medicine, Fondazione IRCCS (Istituto Di Ricovero e Cura a Carattere Scientifico) Istituto Nazionale Tumori (INT), Via Giacomo Venezian 1, 20133 Milan, Italy; 56 Familial Cancer Centre, Peter MacCallum Cancer Centre, Locked Bag 1, A’Beckett Street, Melbourne, VIC 8006 Australia; 57 Sir Peter MacCallum Department of Oncology, University of Melbourne, Parkville, VIC 3052 Australia; 58 Immunology and Molecular Oncology Unit, Veneto Institute of Oncology IOC - IRCCS, Via Gattamelata 64, Padua, Italy; 59 Department of Population Sciences, Beckman Research Institute of City of Hope, Duarte, CA USA; 60 Department of Obstetrics and Gynecology, University of Helsinki and Helsinki University Hospital, Biomedicum Helsinki, P.O. BOX 700, (Haartmaninkatu 8), 00029 HUS Helsinki, Finland; 61 Department of Gynaecology and Obstetrics, University Hospital Düsseldorf, Heinrich-Heine University Düsseldorf, Düsseldorf, Germany; 62 513 Parnassus Ave., HSE 901E, San Francisco, CA 94143-0794 USA; 63 Clinical Genetics Research Laboratory, Department of Medicine, Cancer Biology and Genetics, Memorial Sloan-Kettering Cancer Center, 1275 York Avenue, New York, NY 10044 USA; 64 Department of Molecular Genetics, National Institute of Oncology, Budapest, Hungary; 65 5841 South Maryland Avenue, MC 2115 Chicago, IL USA; 66 Department of Preventive Medicine, Seoul National University College of Medicine, 103 Daehak-ro, Jongno-gu, Seoul, 110-799 Korea; 67 NRG Oncology, Statistics and Data Management Center, Roswell Park Cancer Institute, Elm St & Carlton St, Buffalo, NY 14263 USA; 68 Unit of Molecular Bases of Genetic Risk and Genetic Testing, Department of Preventive and Predicted Medicine, Fondazione IRCCS (Istituto Di Ricovero e Cura a Carattere Scientifico) Istituto Nazionale Tumori (INT), c/o Amaedeolab, via GA Amadeo 42, 20133 Milan, Italy; 69 Department of OB/GYN, Medical University of Vienna, Waehringer Guertel 18-20, A 1090 Vienna, Austria; 70 Department of Epidemiology, Netherlands Cancer Institute, P.O. Box 90203, 1000 BE Amsterdam, The Netherlands; 71 Department of Medical Oncology, Family Cancer Clinic Erasmus University Medical Center Cancer institute, P.O. Box 5201, 3008 AE Rotterdam, The Netherlands; 72 Genomics Center, Centre Hospitalier Universitaire de Québec Research Center and Laval University, 2705 Laurier Boulevard, Quebec City, Quebec Canada; 73 Department of OB/GYN and Comprehensive Cancer Center, Medical University of Vienna, Waehringer Guertel 18-20, A 1090 Vienna, Austria; 74 Genetic Epidemiology Laboratory, Department of Pathology, University of Melbourne, Parkville, Victoria Australia; 75 Service de Génétique Oncologique, Institut Curie, 26, rue d’Ulm, Paris, Cedex 05, France; 76 National Human Genome Research Institute, National Institutes of Health Building 50, Room 5312, 50 South Drive, MSC 004, Bethesda, MD 20892-8004 USA; 77 Section of Genetic Oncology, Department of Laboratory Medicine, University and University Hospital of Pisa, Pisa, Italy; 78 Department of Genetics, Portuguese Oncology Institute, Rua Dr. António Bernardino de Almeida, 4200-072 Porto, Portugal; 79 Cancer Research Initiatives Foundation, Sime Darby Medical Centre, 1 Jalan SS12/1A, Subang Jaya, 47500 Malaysia; 80 University Malaya Cancer Research Institute, University Malaya, 50603 Kuala Lumpur, Malaysia; 81 Department of Epidemiology, Columbia University, New York, NY USA; 82 Department of Clinical Genetics, Odense University Hospital, Sonder Boulevard 29, Odense C, Denmark; 83 Latvian Biomedical Research and Study Centre, Ratsupites str 1, Riga, Latvia; 84 Program in Cancer Genetics, Departments of Human Genetics and Oncology, McGill University, Montreal, Quebec Canada; 85 Divison of Human Cancer Genetics, Departments of Internal Medicine and Molecular Virology, Immunology and Medical Genetics, Comprehensive Cancer Center, The Ohio State University, 998 Biomedical Research Tower, Columbus, OH USA; 86 Department of Medical Oncology, Beth Israel Deaconess Medical Center, 330 Brookline Avenue, Boston, MA 02215 USA; 87 Cancer Genetics Laboratory, Department of Genetics, University of Pretoria, Private Bag X323, Arcadia, 0007 South Africa; 88 Clinical Cancer Genetics Laboratory, Memorial Sloane Kettering Cancer Center, New York, NY USA; 89 Department of Gynaecology and Obstetrics, University Hospital Ulm, Ulm, Germany; 90 Clinical Cancer Genetics, City of Hope, 1500 East Duarte Road, Duarte, California 91010 USA; 91 Institute of Oncology, Rivka Ziv Medical Center, 13000 Zefat, Israel; 92 The Faculty of Medicine, Bar-Ilan University, Zefat, Israel; 93 4301 West Markham Street, Slot 793, Little Rock, AR 72205 USA; 94 Present Address: School of Women’s and Children’s Health, University of New South Wales and The Kinghorn Cancer Centre, Garvan Institute of Medical Research, 384 Victoria Street, Darlinghurst, NSW 2010 Australia

**Keywords:** Hereditary breast and ovarian cancer, Transheterozygosity, BRCA1, BRCA2

## Abstract

**Background:**

Most *BRCA1* or *BRCA2* mutation carriers have inherited a single (heterozygous) mutation. Transheterozygotes (TH) who have inherited deleterious mutations in both *BRCA1* and *BRCA2* are rare, and the consequences of transheterozygosity are poorly understood.

**Methods:**

From 32,295 female *BRCA1/2* mutation carriers, we identified 93 TH (0.3 %). “Cases” were defined as TH, and “controls” were single mutations at *BRCA1* (SH1) or *BRCA2* (SH2). Matched SH1 “controls” carried a BRCA1 mutation found in the TH “case”. Matched SH2 “controls” carried a BRCA2 mutation found in the TH “case”. After matching the TH carriers with SH1 or SH2, 91 TH were matched to 9316 SH1, and 89 TH were matched to 3370 SH2.

**Results:**

The majority of TH (45.2 %) involved the three common Jewish mutations. TH were more likely than SH1 and SH2 women to have been ever diagnosed with breast cancer (BC; *p* = 0.002). TH were more likely to be diagnosed with ovarian cancer (OC) than SH2 (*p* = 0.017), but not SH1. Age at BC diagnosis was the same in TH vs. SH1 (*p* = 0.231), but was on average 4.5 years younger in TH than in SH2 (*p* < 0.001). BC in TH was more likely to be estrogen receptor (ER) positive (*p* = 0.010) or progesterone receptor (PR) positive (*p* = 0.013) than in SH1, but less likely to be ER positive (*p* < 0.001) or PR positive (*p* = 0.012) than SH2. Among 15 tumors from TH patients, there was no clear pattern of loss of heterozygosity (LOH) for *BRCA1* or *BRCA2* in either BC or OC.

**Conclusions:**

Our observations suggest that clinical TH phenotypes resemble SH1. However, TH breast tumor marker characteristics are phenotypically intermediate to SH1 and SH2.

**Electronic supplementary material:**

The online version of this article (doi:10.1186/s13058-016-0768-3) contains supplementary material, which is available to authorized users.

## Background

Women who have inherited mutations in *BRCA1* or *BRCA2* are at greatly increased risk of developing breast cancer (BC) and ovarian cancer (OC) [25, 38]. Identification of a mutation at these loci can lead to risk or mortality reduction if optimal surveillance, risk-reducing mastectomy (RRM), and risk-reducing salpingo-oophorectomy (RRSO) are applied [8, 29]. In addition, treatment of cancers in mutation carriers has advanced with the development of PARP inhibitors, which take advantage of the loss of *BRCA1/2* function in tumors [37]. *BRCA1* and *BRCA2* are tumor suppressor genes, and tumors from the majority of mutation carriers have loss of heterozygosity (LOH), with loss of the normal allele, so there is no functioning protein [6, 7, 13, 31]. In early studies, including a small number of tumor samples obtained from large BC and OC families, it was suggested that greater than 85 % of *BRCA1-* or *BRCA2*-associated cancers exhibited LOH, and all showed loss of the normal allele.

The vast majority of *BRCA1* and *BRCA2* mutation carriers are single heterozygotes for *BRCA1* (SH1) or *BRCA2* (SH2). Homozygosity of missense alleles at *BRCA2* (*FANCD1*) leads to Fanconi Anemia and increased cancer susceptibility, notably hematological malignancies [15, 22]. At least three Fanconi Anemia cases are attributable to *BRCA2/FANCD1* homozygous mutations [22]. Observations of homozygosity or compound heterozygosity at *BRCA1* are very rare. Domchek et al. [9] reported a female patient with short stature, microcephaly, developmental delay, significant toxicity from chemotherapy, and epithelial ovarian carcinoma diagnosed at age 28 years. This woman was a compound heterozygote at *BRCA1*, with mutations c.2457delC (p.Asp821Ilefs*25) and c.5207 T > C (p.Val1736Ala). Both of these mutations are likely to be deleterious variants in *BRCA1*-associated cancer. The only other reported case of biallelic *BRCA1* mutations was in a woman with multiple congenital anomalies consistent with a Fanconi anemia-like disorder and breast cancer at age 23 [30].

Transheterozygosity (TH) is the state of heterozygosity at two different loci. Here, we define TH to be inheritance of deleterious mutations in both *BRCA1* and *BRCA2*. Reports on several *BRCA1/2* transheterozygotes (TH) have been reported in the literature, mainly without further details on tumor or patient phenotype. Ramus et al. [27] reported on one TH who had been diagnosed with both BC and OC, and was identified as having a mutation in *BRCA1* c.68_69delAG (185/187delAG) and *BRCA2* c.5946delT (6174delT). LOH in these tumors was not found. Additional reports identified TH for *BRCA1* c.2389G > T and *BRCA2* c.3068dupA [21], *BRCA1* c.68_69delAG and a *BRCA2* c.5946delT [36], and TH with *BRCA1* c.68_69delAG and *BRCA2* c.5946delT [11] in four cases. In addition, a number of reports of TH with LOH in cancer samples have been published. Randall et al. [28] reported one TH identified with a *BRCA1* c.3770_3771delGA and *BRCA2* c.5946delT, and being affected with both BC and OC. For the BC, only LOH at the *BRCA1* locus was found (not at *BRCA2)*, and the OC sustained LOH at both *BRCA1* and *BRCA2*. Tesoriero et al. [35] reported a TH with *BRCA1* c.3770_3771delGA and *BRCA2* c.5946delT. The BC of this patient lost the wild-type *BRCA2* allele. Bell et al. [1] reported on a TH with c.5266dupC *BRCA1* and c.5946delT *BRCA2* mutation having three independent BCs. They showed that LOH occurred in two *BRCA2* and one *BRCA1* tumor. A large clinic-based series of 1191 carriers from Israel [20] identified 16 TH females, 14 with the c.68_69delAG *BRCA1* and c.5946delT *BRCA2* mutations and two with the c.5266dupC *BRCA1* and c.5946delT *BRCA2* mutations. A study from Germany identified eight female TH from 8162 BC/OC families and compared the clinical characteristics of the TH to their SH relatives and to SH in the family-based study [14].

To characterize the nature of TH and clinical phenotypes of TH, we used the Consortium of Investigators of Modifiers of *BRCA1/2* (CIMBA) dataset of 32,295 female *BRCA1/2* mutation carriers ascertained in high-risk clinics and population-based studies. From this dataset, we investigated the occurrence of TH, we compared the characteristics and features of BC and OC in TH and single *BRCA1* or *BRCA2* mutations, and we examined LOH in as many cancer samples as possible.

## Methods

### Study sample

Details of CIMBA participating centers and data collection have been reported previously [5]. All the included mutation carriers participated in clinical and research studies at the host institutions after providing informed consent under IRB-approved protocols. Fifty-five centers and multicenter consortia (Additional file 1: Table S1) submitted data that met the CIMBA inclusion criteria [5]. Only female carriers with pathogenic *BRCA1/2* mutations, concerning TH, SH1, and SH2 mutation carriers, were included in the current analysis. Pathogenicity of mutation was defined as follows: 1) generating a premature termination codon (PTC), except variants generating a PTC in exon 27 after codon 3010 of *BRCA2*; 2) large in-frame deletions that span one or more exons; and 3) deletion of transcription regulatory regions (promoter and/or first exon) expected to cause lack of expression of mutant allele. We also included missense variants considered pathogenic by using multifactorial likelihood approaches [4, 12]. Mutations that did not meet the above criteria but have been classified as pathogenic by Myriad Genetics, Inc. (Salt Lake City, UT, USA) were also included.

Mutations are described using the Human Genome Variation Society (HGVS) nomenclature (http://www.HGVS.org/varnomen) where the nucleotide numbering is from the A of the ATG translation initiator codon. For deletions or insertions, the most 3′ position possible was arbitrarily assigned as the altered nucleotide. The description of mutations of all types is given at the genomic level (using cDNA reference sequences NM_007294.3/*BRCA1* and NM_000059/*BRCA2*). BIC nomenclature was also presented for common variants that are familiar to many researchers and clinicians by their BIC designation (http://research.nhgri.nih.gov/bic). For BIC nomenclature, cDNA sequences were used as reference sequence (Genbank: U14680/*BRCA1* and NM_000059.1/*BRCA2*). The nucleotide numbering is from nucleotide 1 of the cDNA gene sequence and for deletions or insertions the most 3′ position possible was arbitrarily assigned as the altered nucleotide.

In order to compare the TH with SH1 and SH2 mutation carriers on phenotypes of interest, we created a matched case–control set, in which “cases” were defined as TH, and “controls” were SH1 and SH2 mutation carriers. Matched SH1 “controls” carried a BRCA1 mutation found in the TH “case”. Matched SH2 “controls” carried a BRCA2 mutation found in the TH “case”. SH1 and SH2 were not matched to TH for any other characteristics. Using this approach, we identified 91 TH and 9316 matched SH1 mutation carriers, and 89 TH and 3370 matched SH2 mutation carriers.

### Loss of heterozygosity

From 10 TH individuals, tumor tissue was available from twelve tumors, and blood DNA from 10 TH. From one case, tumor tissue from both BC and OC was available, and from another case affected with bilateral BC, tumor samples were available from both breast tumors. Hematoxylin and eosin (H&E) slides from each tumor were examined by a specialist pathologist. Areas of >80 % tumor cells were marked for macro-dissection. DNA from two 10-micron unstained slides was extracted using the Qiagen QIAmp DNA FFPE Tissue Kit using the standard protocol but with 500 μl deparaffinization solution.

We performed micro-satellite analysis to objectively detect LOH as described previously [16]. We amplified patient tumor and blood DNA for two markers within *BRCA1* (D17S855 and D17S1322) and four markers around *BRCA2* (D13S290, D13S260, D13S1698, and D13S171). The heterozygosity for these markers ranged from 0.46 to 0.82 [17, 26]. Primer sequences and distance from *BRCA1* or *BRCA2* are given in Additional file 1 (Table S2). After polymerase chain reaction (PCR) amplification, samples were size-separated on a 96 capillary DNA analyzer (Applied Biosystems 3730xl). Data were analyzed using Genemapper Software (Applied Biosystems). For micro-satellites that were heterozygous, the ratios of allele peak heights for each tumor sample were compared to the allele peak heights for the blood DNA sample using the following formula L = (at2 X an1)/(at1 X an2), where L = the ratio; a = the height of the peak; n1 and n2 = normal allele 1 and normal allele 2; t1 and t2 = tumor allele 1 and tumor allele 2. All ten cases were informative for at least one marker in *BRCA1*. Where cases were informative for both markers, the LOH data were consistent for the two nearby markers. All ten cases were also informative for at least one of the four markers in *BRCA2*. In two cases, the data were not consistent across all markers in the 1.74 MB region and the data for the marker closest to *BRCA2* was used.

To complement the information obtained from micro-satellite analysis, we also undertook DNA sequence analysis. For each individual, a small region (<200 bp) around each of their two mutations was PCR-amplified from both tumor and blood DNA. DNA from peripheral blood of a healthy control individual was also amplified for each fragment as a control for no mutation. We used 10 ng of DNA in the PCR reaction, using standard protocol and primer sequences (given in Additional file 1: Table S3). All three samples for each mutation were then treated with EXO-SAP-IT (Affymetrix) and Sanger sequenced using standard methods [32]. This sequencing was used to confirm the presence of each mutation in the blood DNA from the patient and not in the control sample. We also assessed the mutation status in the tumor to determine if LOH had occurred. Since we extracted areas of >80 % tumor cells, both alleles can be present even when LOH is present, due to contaminating normal tissue. Therefore, for each tumor we determined for each mutation if the two alleles were at an equal ratio compared to the germline sample or if there was a decrease in one of the two alleles.

### Statistical Analysis

For comparison of TH and SH mutation carriers, contingency table analysis using a chi-square test was used for dichotomous variables, and a *t* test for continuous variables. Fisher’s exact tests were used if sample sizes in any contingency table cell were less than five. Analyses were done in STATA, v. 13.1.

## Results

### Characteristics of TH versus SH1 and SH2 mutation carriers

**Fig. 1:**
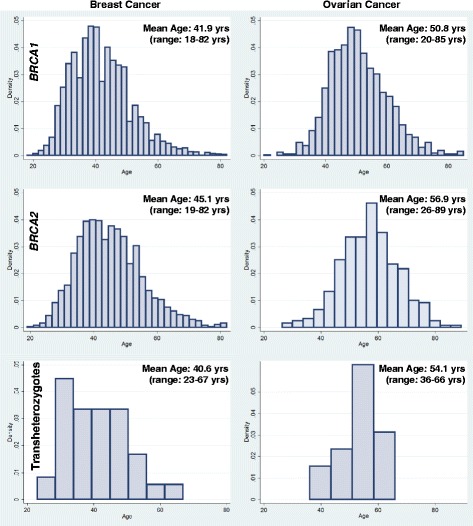
Age of breast and ovarian cancer diagnosis by mutation status

Table 1 describes the 93 female TH from 84 families identified from the CIMBA database. Among the matched TH-SH1/SH2 sets, 25 had no cancer diagnosis. The average age of these women was 39 years and the average age at diagnosis of BC was 41 years. Only 16 women were less than age 41 and 9 women were over age 41 at the time of diagnosis (mean age 49.9, range 41.4–67.9). Table 2 shows that OC age for the matched BRCA1 TH cases was 51.1 years and SH1 controls was 50.9 years (*p* = 0.154). For the matched BRCA2 set the average OC age for the TH cases was 54.7 years and for SH2 controls was 56.8 years (*p* = 0.421) (Fig. 1).

**Table 1:** Transheterozygote *BRCA1 + BRCA2* mutations in 93 women

*BRCA1* mutation	*BRCA2* mutation	*N*	%	Breast cancer only		Ovarian cancer only		Breast + ovarian cancer		No cancer		Self-identified race/ethnicity	Country of ascertainment
HGVS: genomic level	HGVS: genomic level												
				*n*	%	*n*	%	*n*	%	*n*	%		
c.-19-?_80 + ?dup	c.8633-?_8754 + ?amp*	1	1.1	1	1.1	0	0.0	0	0.0	0	0.0	Jewish	Hungary
c.68_69delAG	c.5946delT	31	33.3	13	13.9	1	1.1	3	3.2	14	15.0	Caucasian, Jewish, NR	USA, Hungary, Israel
c.68_69delAG	c.5722_5723delCT	1	1.1	1	1.1	0	0.0	0	0.0	0	0.0	Caucasian	Germany
c.1016delA	c.7379_7382delACAA	1	1.1	1	1.1	0	0.0	0	0.0	0	0.0	Asian	USA
c.1390delA*	c.658_659delGT	1	1.1	1	1.1	0	0.0	0	0.0	0	0.0	Hispanic	USA
c.1504_1508del5	c.2798_2799delCA	1	1.1	1	1.1	0	0.0	0	0.0	0	0.0	Asian	Korea
c.1504_1508del5	c.462_463delAA	1	1.1	0	0.0	0	0.0	1	1.1	0	0.0	Caucasian	Germany
c.1687C > T	c.6469C > T	1	1.1	0	0.0	0	0.0	1	1.1	0	0.0	Caucasian	Italy
c.1793 T > ?	c.8537_8538delAG	1	1.1	0	0.0	0	0.0	1	1.1	0	0.0	Caucasian	USA
c.181 T > G	c.1318_1319dupCT	3	3.2	1	1.1	0	0.0	0	0.0	2	2.2	Caucasian	Austria
c.211A > G	c.4380_4381delTT	1	1.1	1	1.1	0	0.0	0	0.0	0	0.0	Caucasian	UK
c.212 + 1G > A	c.739_740delAT*	1	1.1	1	1.1	0	0.0	0	0.0	0	0.0	Caucasian	Spain
c.213-12A > G	c.7180A > T	1	1.1	1	1.1	0	0.0	0	0.0	0	0.0	Caucasian	Italy
c.2389G > T	c.3068dupA	1	1.1	1	1.1	0	0.0	0	0.0	0	0.0	Caucasian	Canada
c.2405_2406delTG	c.4284dupT	1	1.1	0	0.0	0	0.0	1	1.1	0	0.0	Caucasian	Italy
c.246delT	c.517-2A > G	2	2.2	1	1.1	0	0.0	0	0.0	1	1.1	Caucasian	UK
c.301 + 1G > A	c.5682C > G	1	1.1	1	1.1	0	0.0	0	0.0	0	0.0	Caucasian	USA
c.3048_3052dup5	c.2830A > T	2	2.2	1	1.1	0	0.0	0	0.0	1	1.1	NR	Sweden
c.3155delA	c.3160_ 3163delGATA	2	2.2	1	1.1	0	0.0	0	0.0	1	1.1	Caucasian	Australia
c.3196G > T*	c.658_659delGT	1	1.1	1	1.1	0	0.0	0	0.0	0	0.0	Caucasian	Germany
c.3228_3229delAG	c.3689delC	1	1.1	0	0.0	0	0.0	0	0.0	1	1.1	Caucasian	UK
c.3228_3229delAG	c.9253dupA	1	1.1	1	1.1	0	0.0	0	0.0	0	0.0	Caucasian	Italy
c.3400G > T	c.2808_2811delACAA	2	2.2	1	1.1	0	0.0	0	0.0	1	1.1	Caucasian	UK
c.3477_3480 delAAAG	c.9401delG	1	1.1	0	0.0	1	1.1	0	0.0	0	0.0	Caucasian	Italy
c.3627dupA	c.6724_6725delGA	1	1.1	1	1.1	0	0.0	0	0.0	0	0.0	Asian	Korea
c.3700_3704del5	c.681 + 1G > A	1	1.1	1	1.1	0	0.0	0	0.0	0	0.0	Caucasian	Australia
c.3700_3704del5	c.1815dupA	1	1.1	1	1.1	0	0.0	0	0.0	0	0.0	Caucasian	Germany
c.3756_3759delGTCT	c.7757G > A	1	1.1	1	1.1	0	0.0	0	0.0	0	0.0	Caucasian	USA
c.3759_3760delTA	c.9699_9702 delTATG	1	1.1	0	0.0	0	0.0	1	1.1	0	0.0	Hispanic	USA
c.3770_3771delAG	c.5946delT	2	2.2	1	1.1	0	0.0	1	1.1	0	0.0	NR, Jewish	Australia, USA
c.3839_3843 delinsAGGC	c.1636delT	2	2.2	0	0.0	1	1.1	0	0.0	1	1.1	NR	France
c.390C > A	c.3018delA	1	1.1	1	1.1	0	0.0	0	0.0	0	0.0	Asian	Korea
3910delG	c.2830A > T	1	1.1	1	1.1	0	0.0	0	0.0	0	0.0	Caucasian	Germany
c.3916_3917delTT	c.5380delG*	1	1.1	0	0.0	0	0.0	1	1.1	0	0.0	Caucasian	Italy
c.4035delA	c.658_659delGT	1	1.1	0	0.0	0	0.0	0	0.0	1	1.1	Caucasian	Australia
c.4065_4068delTCAA	c.5350_5351delAA	1	1.1	1	1.1	0	0.0	0	0.0	0	0.0	Caucasian	USA
c.4186-?_4357 + ?dup	c.2636_2637delCT	2	2.2	1	1.1	0	0.0	0	0.0	1	1.1	Caucasian	UK
c.427G > T	c.8730delT	1	1.1	1	1.1	0	0.0	0	0.0	0	0.0	Caucasian	Denmark
c.5030_5033 delCTAA	c.1399A > T	1	1.1	1	1.1	0	0.0	0	0.0	0	0.0	Asian	Korea
c.5123C > A	c.6275_6276delTT	1	1.1	1	1.1	0	0.0	0	0.0	0	0.0	Caucasian	Germany
c.5136G > A	c.4965delC	1	1.1	1	1.1	0	0.0	0	0.0	0	0.0	Asian	USA
c.5193 + 1delG	c.658_659delGT	1	1.1	0	0.0	1	1.1	0	0.0	0	0.0	Caucasian	Germany
c.5251C > T	c.6753_6754delTT	1	1.1	1	1.1	0	0.0	0	0.0	0	0.0	Caucasian	Austria
c.5266dupC	c.8364G > A	1	1.1	1	1.1	0	0.0	0	0.0	0	0.0	Caucasian	Austria
c.5266dupC	c.5946delT	5	5.4	3	3.2	0	0.0	1	1.1	1	1.1	Jewish	UK, Israel
c.5266dupC	c.4478_4481delAAAG	1	1.1	1	1.1	0	0.0	0	0.0	0	0.0	Caucasian	Germany
c.5266dupC	c.5645C > A	1	1.1	0	0.0	0	0.0	1	1.1	0	0.0	Caucasian	Germany
c.5406 + 664_*8273del	c.9748dupT	1	1.1	1	1.1	0	0.0	0	0.0	0	0.0	Caucasian	Greece
c.548-?_4185 + ?del	c.2269A > T*	1	1.1	0	0.0	0	0.0	1	1.1	0	0.0	Caucasian	Germany
c.962G > A	c.2231C > G	1	1.1	1	1.1	0	0.0	0	0.0	0	0.0	Caucasian	Germany
Total		93	100	51	54.8	4	4.3	13	14.0	25	26.9		
Mean age (range)				39.9 (23–67)		59.2 (57–62)		41.9 (26–53)		39.1 (20–68)			

*Not included in the matched analysis because one of the mutations found in the TH was not found among the SH1/SH2 carriers

*HGVS* Human Genome Variation Society, *NR* not reported

**Table 2:** Description of *BRCA1, BRCA2*, and transheterozygote *BRCA1 + BRCA2* mutation carriers

Variable	Value	*BRCA1 + BRCA2 (TH)N(%)*	*BRCA1 (SH1)N(%)*	*P* value*	*P* value*	*BRCA1* + *BRCA2 (TH) N(%)*	*BRCA2 (SH2)N(%)*	*P* value**	*P* value**
Total matched		91	9316			89	3370		
Year of birth	<1940 1941–1950 1951–1960 1961–1970 >1970	5 (5.5) 20 (22.0) 21 (22.6) 27 (29.0) 18 (19.8)	886 (9.5) 1628 (17.4) 2607 (28.0) 2409 (25.9) 1779 (19.0)	0.424	(ref) 0.112 0.474 0.153 0.245	5 (5.6) 19 (21.3) 19 (21.3) 27 (30.3) 19 (21.3)	486 (14.4) 735 (21.8) 914 (27.1) 724 (21.5) 511 (15.2)	**0.025**	(ref) 0.060 0.156 **0.005** **0.007**
Ethnicity	White African American Asian Hispanic Jewish Other	47 (51.6) 0 (0) 6 (6.6) 1 (1.1) 30 (33.0) 7 (7.7)	5736 (61.6) 20 (0.2) 82 (0.9) 143 (1.5) 1779 (19.1) 1556 (16.7)	**<0.001**	(ref) 1.00* **<0.001** 1.00 **0.002** --	45 (50.6) 0 (0) 6 (6.7) 2 (2.2) 29 (32.6) 7 (7.9)	1686 (50.0) 15 (0.4) 66 (2.0) 57 (1.7) 936 (27.8) 610 (18.1)	**0.007**	(ref) 1.00* **0.004** 0.667 0.573 **--**
Breast cancer	No Yes	29 (31.9) 62 (68.1)	4470 (48.0) 4846 (52.0)	**0.002**		29 (32.6) 60 (67.4)	1671 (49.6) 1699 (50.4)	**0.002**	
Age of breast cancer	Mean (range)	40.4 (23–67)	41.9 (18–82)	0.231		40.5 (23–67)	45.0 (19–82)	**<0.001**	
Ovarian cancer	No Yes	74 (81.3) 17 (18.7)	7766 (83.4) 1550 (16.6)	0.603		74 (83.1) 15 (16.9)	3056 (90.7) 314 (9.3)	**0.017**	
Age of ovarian cancer	Mean (range)	54.1 (36–66)	50.9 (20–85)	0.154		54.7 (42–66)	56.8 (26–89)	0.421	
Bilateral mastectomy	No Yes	58 (63.7) 8 (9.0)	4807 (51.6) 809 (8.7)	0.599		58 (65.2) 8 (9.0)	1856 (55.0) 305 (9.1)	0.646	
Prophylactic oophorectomy	No Yes	45 (49.5) 24 (26.4))	3583 (38.4) 2476 (26.6)	0.307		45 (50.6) 24 (27.0)	1388 (41.2) 980 (29.1)	0.272	
Follow up age (if no cancer)	Mean (range)	39.1 (20–68)	40.5 (18–99)	0.587		39.1 (20–68)	44.1 (18–94)	0.068	

*Matched *BRCA1* mutation carriers vs *BRCA1 + BRCA2* mutation carriers; **matched *BRCA2* mutation carriers vs *BRCA1 + BRCA2* mutation carriers

Significant *p* values are shown in bold type

The most common TH involved inheritance of two of the three common Jewish mutations: 5 (5.4 %) women inherited *BRCA1* c.5266dupC and *BRCA2* c.5946delT; 31 (33.3 %) women inherited *BRCA1* c.68_69delAG and *BRCA2* c.5946delT. Six (6.5 %) women carried one of the three common Jewish mutations and another mutation. The majority of the remaining TH were observed only once. The majority of the TH self-identified as non-Hispanic Caucasian or Jewish. Of the 6907 women who carried one of the Jewish founder mutations, 2732 (39.6 %) self-identified as Jewish, 947 (13.7 %) were unknown, and 3225 (46.7 %) reported an ethnicity other than Jewish. We observed two TH in Hispanics and six TH in Asians (four of which were Korean). Of the 93 TH, 51 were diagnosed with BC only, 4 with OC only, 13 with both BC and OC, and 25 with no cancer diagnosis.

The matched datasets included 91 TH and 9316 SH1 for the *BRCA1* matched analysis, and 89 TH and 3370 SH2 for the *BRCA2* matched analysis. Two *BRCA1* mutations were observed among the TH in our dataset that were not observed among SH1 (c.1390delA and c.3196G > T), and four *BRCA2* mutations were observed in the TH dataset that were not observed among the SH2 (c.8633-?_8754 + ?amp, c.739_740delAT, c.5380delG, and c.2269A > T). These six carriers were not included in the analysis (denoted by asterisk in Table 1). TH were more likely to be born more recently (i.e., since 1961) than SH2 mutation carriers but not when compared to SH1s (Table 2). The TH group consisted of more individuals from Asian ancestry compared to the SH1 and SH2 groups, with an excess of women having a Jewish ancestry vs. the SH1 group. TH were more likely to have ever been diagnosed with BC than SH1 or SH2 individuals (68.1 % vs. 52.0 %; *p* = 0.002, and 67.4 % vs. 50.4 %; *p* = 0.002), and TH were more likely to be diagnosed with OC than SH2 women (16.9 % vs. 9.3 %; *p* = 0.017), which was not observed in TH vs. SH1 women, perhaps due to the lower incidence of OC in *BRCA2* vs. *BRCA1*. Age at BC diagnosis was significantly different for TH vs. SH2 (40.5 years vs. 45.0 years; *p* < 0.001), but there was no difference between TH and SH1.

There were 64 TH cases with BC. Of these, 62 TH were matched to 4846 SH1s and 60 TH were matched to 1699 SH2 (Table 3). TH were more likely to have estrogen receptor (ER)- and progesterone receptor (PR)-positive BC than SH1s (ER: 42.9 % vs. 24.0 %; *p* = 0.010; PR: 40.6 % vs. 20.0 %; *p* = 0.013). In contrast, the BCs of TH were less likely ER- and PR-positive than in SH2s (ER: 42.9 % vs. 76.5 %; *p* < 0.0001; PR: 40.6 % vs. 62.8 %; *p* = 0.012). The proportion of ER- and PR-positive BCs in TH was intermediate to that of SH1 and SH2. No difference was seen regarding the HER2 status between the BCs of TH and SH1s and SH2s, respectively, although the available numbers were small. No differences in other BC characteristics (morphology, grade, stage) were observed.

**Table 3:** Breast tumor characteristics of *BRCA1, BRCA2,* and transheterozygote *BRCA1 + BRCA2* mutation carriers

Trait	Value	*BRCA1 + BRCA2 (TH) N(%)*	*BRCA1 (SH1) N(%)*	*P* value	*BRCA1 + BRCA2 (TH) N(%)*	*BRCA2 (SH2) N(%)*	*P* value
*N*		62	4846		60	1699	
HER2	Negative	14 (93.3)	908 (88.7)	1.00	15 (93.8)	274 (86.2)	0.706
	Positive	1 (7.7)	116 (11.3)		1 (6.3)	44 (13.8)	
PR	Negative	19 (59.4)	1260 (80.0)	**0.013**	19 (59.4)	215 (37.2)	**0.012**
	Positive	13 (40.6)	356 (20.0)		13 (40.6)	363 (62.8)	
ER	Negative	20 (57.1)	1347 (76.0)	**0.010**	20 (57.1)	150 (23.5)	**<0.0001**
	Positive	15 (42.9)	424 (24.0)		15 (42.9)	487 (76.5)	
Nodal status	Negative	20 (66.7)	1197 (65.1)	0.854	19 (65.5)	399 (61.3)	0.647
	Positive	10 (33.3)	643 (35.0)		10 (34.5)	252 (38.7)	
Grade	Well differentiated	2 (7.1)	36 (2.3)	0.161	2 (7.1)	36 (6.4)	0.690
	Moderately differentiated	8 (28.8)	342 (22.1)		8 (28.8)	207 (36.6)	
	Poorly/undifferentiated	18 (64.3)	1172 (75.6)		18 (64.3)	322 (57.0)	
Stage	0	1 (4.8)	34 (3.6)	0.541	1 (4.6)	48 (13.9)	0.065
	1	7 (33.3)	399 (42.2)		7 (31.8)	123 (35.7)	
	2	13 (61.9)	440 (46.6)		14 (63.6)	124 (35.9)	
	3	0 (0)	65 (6.9)		0 (0)	36 (10.4)	
	4	0 (0)	7 (0.7)		0 (0)	14 (4.1)	
Morphology	Ductal	26 (70.3)	1544 (74.3)	0.345	27 (73.0)	629 (78.8)	0.359
	Lobular	3 (8.1)	61 (2.9)		3 (8.1)	70 (8.8)	
	Medullary	3 (8.1)	173 (8.3)		2 (5.4)	13 (1.6)	
	Other	5 (13.5)	301 (14.5)		5 (13.5)	86 (10.8)	
Number of positive nodes (SD)		2 (6.1)	1.2 (3.4)	0.215	2.1 (6.2)	1.7 (3.9)	0.627
Tumor size (SD)		19.0 (14.9)	18.3 (12.5)	0.775	19.0 (14.9)	19.2 (14.6)	0.932

Significant *p* values are shown in bold type

*ER* estrogen receptor, *PR* progesterone receptor, *SD* standard deviation

Only 17 TH were diagnosed with OC, and thus we had limited data on features of OC to make inferences regarding differences in TH compared with SH1 or SH2. No statistically significant differences were observed for OC traits between TH and SHs (Table 4). Surprisingly, four borderline tumors were reported in both the SH1 and SH2 groups.

**Table 4:** Ovarian tumor characteristics of *BRCA1, BRCA2,* and transheterozygote *BRCA1 + BRCA2* mutation carriers

Trait	Value	*BRCA1 + BRCA2 (TH)N(%)*	*BRCA1 (SH1)N(%)*	*P* value	*BRCA1 + BRCA2 (TH)﻿N(%)*	*BRCA2 (SH2)N(%)*	*P* value
*N*		17	1550		15	314	
Grade	Well differentiated	0	8 (2.8)	0.930	0	4 (6.2)	0.847
	Moderately differentiated	1 (25)	60 (20.8)		1 (25)	12 (18.5)	
	Poorly/undifferentiated	3 (75)	220 (76.4)		3 (75)	49 (75.4)	
Stage	1	0	39 (17.4)	0.600	0	6 (13.3)	0.589
	2	1 (33.3)	31 (13.8)		1 (33.3)	5 (11.1)	
	3	2 (66.7)	120 (53.6)		2 (66.7)	28 (62.2)	
	4	0	34 (15.2)		0	6 (13.3)	
Morphology	Serous	5 (83.3)	292 (66.8)	0.905	5 (83.3)	71 (73.2)	0.943
	Mucinous	0	4 (0.9)		0	2 (2.0)	
	Endometroid	0	44 (10.1)		0	7 (7.2)	
	Clear cell	0	6 (1.4)		0	2 (2.0)	
	Other	1 (16.7)	91 (20.8)		1 (16.7)	15 (15.5)	
Behavior	Invasive	7 (100)	449 (99.1)	0.803	6 (100)	89 (95.7)	0.604
	Borderline	0	4 (0.9)		0	4 (4.3)	

### Loss of heterozygosity

Due to the frequent LOH in SH individuals, we examined the hypothesis that either *BRCA1* or *BRCA2* would be lost in each of the TH individuals due to LOH, and that whichever gene was lost could have an impact on their tumor characteristics. Of the 68 TH individuals with cancer, LOH analysis of three tumors from two cases had previously been published by our group using the same methods as the newly identified cases [27]. In the context of the current study, 12 additional tumor samples from 10 patients were analyzed (Table 5). We first used micro-satellite markers and an objective ratio of peak heights to determine if there was loss of one of the alleles when an individual was heterozygous [3] (Additional file 1: Tables S4 and S5). LOH analysis with micro-satellite markers normally includes linkage or segregation data to determine if the normal allele is lost. Since we did not have samples from other family members, we performed Sanger sequencing at the position of the mutations in both germline and tumor samples to determine which allele was lost. One sample failed for the sequencing so it was not possible to determine whether the normal or mutated allele was lost. Some samples showed loss of the mutant allele, which would suggest random loss. Tumors that exhibited LOH by micro-satellite analysis but did not indicate a decrease of the normal allele by sequencing were not considered to exhibit classic LOH. Following both sets of analyses and including our previously published data, one breast tumor (case 8) and one OC (case 2) showed LOH for *BRCA1*, two breast tumors (cases 9 and 11) showed LOH of *BRCA2*, and the remaining tumors provided no evidence for LOH at either *BRCA1* or *BRCA2* (Table 5).

**Table 5:** Loss of heterozygosity in tumor tissue

					LOH in breast tumor			LOH in ovarian tumor		
Case	Diagnosis	Tissue studied	*BRCA1* mutation	*BRCA2* mutation	Micro-satellite Data	Sequence data	Inference	Micro-satellite data	Sequence data	Inference
1	DCIS	DCIS	c.5136G > A	c.4965delC	*BRCA1, BRCA2*	No	No LOH			
2	Breast	Inv Br	c.1793 T > A	c.8537_8538delAG	*BRCA2*	*BRCA1*	No LOH			
3	Invasive breast	Inv Br	c.68_69delAG	c.5946delT	*BRCA1*	No	No LOH			
5	Invasive breast	DCIS^b^	c.181 T > G	c.1318_1319dupCT	No	*BRCA2*	No LOH			
6 L	Bilateral breast	DCIS^b^	c.5251C > T	c.6753_6754delTT	No	No	No LOH^d^			
6R	Bilateral breast	DCIS^b^	c.5251C > T	c.6753_6754delTT	*BRCA1*	No	No LOH^d^			
7	Invasive breast	DCIS^b^	c.5266dupC	c.8364G > A	No	*BRCA1*	No LOH			
8	Invasive breast	DCIS^b^	c.3700_3704del5	c.681 + 1G > A	*BRCA1, BRCA2*	*BRCA1*	*BRCA1* LOH			
9	Invasive breast	DCIS^b^	c.68_69delAG	c.5946delT	*BRCA2*	*BRCA1, BRCA2*	*BRCA2* LOH			
10	Invasive breast	Inv Br	c.68_69delAG	c.5946delT	*BRCA1, BRCA2*	Failed	Failed^c^			
11^a^	Invasive breast	Inv Br	c.3770_3771delAG	c.5946delT	^a^	^a^	*BRCA2* LOH			
12^a^	Breast	Inv Br	c.68_69delAG	c.5946delT	^a^	^a^	No LOH			
2	Ovary	Ov	c.1793 T > A	c.8537_8538delAG				*BRCA1*	*BRCA1*	*BRCA1* LOH
4	Ovary	Ov	c.68_69delAG	c.5946delT				No	No	No LOH
12^a^	Ovary	Ov	c.68_69delAG	c.5946delT	^a^	^a^	No	^a^	^a^	No LOH

See also Additional file 1 (Table S5)

^a^Previously published, ^b^with micro-invasion, ^c^case failed due to no PCR amplification in the sequencing, ^d^no LOH in either the right or left breast tumor

*DCIS* ductal carcinoma *in situ*, *Inv Br* invasive breast cancer, *LOH* loss of heterozygosity, *Ov* ovarian cancer

## Discussion

This study describes the characteristics of TH compared with SH1 and SH2 mutation carriers and supplements the existing literature regarding LOH in TH. Previously, 35 female TH individuals have been reported in the literature in a series of papers [1, 11, 14, 20, 21, 27, 28, 35, 36]. Only three relatively small studies have so far compared the characteristics of TH to SH women. Lavie et al. [20] reported a non-significant difference in BC occurrence; seven of the 16 TH women (46.7 %) had a personal history of breast carcinoma compared with 372 of 926 (40.2 %) carriers of a single mutation (odds ratio (OR) = 1.3, 95 % confidence interval (CI) 0.4–4.0) [20]. The mean age at diagnosis in TH was 44.6 years, compared with 48.1 in SH. In contrast, Heidemann et al. [14] based on a study of 8 TH individuals suggested that TH develop BC at an earlier age and have more severe disease than those with single heterozygous *BRCA* mutation [14]. Zuradelli et al. [39] reported TH, and provided the possible association between TH and gastric cancer. Similar to the results from the study by Lavie et al. on 16 Ashkenazi Jewish female TH [20], we report that TH were more likely than both SH1 or SH2 to be diagnosed with BC, which was also observed in our series. In addition to prior reports, we observed that TH were more likely to be diagnosed with OC compared with SH2s, but not compared with with SH1s. TH breast tumors were more likely to be ER-/PR-positive than in SH1, but less likely than in SH2 patients, without other different tumor or disease characteristics.

A number of TH had not been diagnosed with cancer by the time this analysis was completed. Twenty-five TH in our cohort had no BC or OC diagnosis at the time of counseling or genotyping. The average age of these TH individuals was 39.1 years (range 20–68). Of these, 16 (64 %) were less than 41 years old at the time of study, which is the average age of BC diagnosis, and 23 (92 %) were younger than the average age of OC diagnosis (54 years) in the CIMBA data. Of these 25 unaffected TH women, 7 (28 %) reported a RRSO compared to 2751 (22.6 %) who underwent RRSO among the total set of SH controls without BC or OC (12,154). Two (8.0 %) cancer-free TH underwent bilateral risk-reducing mastectomy compared to 1076 (8.9 %) SH. In addition, we had missing data for a number of relevant variables that could have impacted some inferences. For example, of the 62 breast cancers in the TH groups, only 21 (34 %) reported stage information.

Although this is the largest series of TH women reported to date, the study is still limited in a number of ways. TH were more likely to be born more recently (i.e., since 1961) than SH2, but not SH1. Since there is evidence that birth cohort may have an important effect on cancer risk [18], the risk associations reported here may require additional evaluation in the future. The higher incidence of BC in the TH group versus both SH1 and SH2 groups, and of OC in the TH vs. the SH2 cohort could be explained by non-random inclusion of TH in the sample, leading to potential biases in associations, and this may limit generalizability of the dataset. Our analyses also do not account for potentially important confounders and the longitudinal nature of the data to follow cancer cases from time of testing to either cancer diagnosis or censoring after risk-reducing surgery. Furthermore, the great majority of missing data on cancer features avoids that certain questions may be appropriately addressed from this type of dataset. Additional future studies are required to completely evaluate these clinically important unresolved issues, and hopefully with the ongoing multinational collaboration within consortia like CIMBA this will be possible in time.

Differences in breast tumor hormone receptor status suggest that TH cases developing BC have an intermediate cancer phenotype between *BRCA1* and *BRCA2*, which would be consistent with the tumors being driven by loss of either *BRCA1* or *BRCA2*. We attempted to determine the frequency of loss of each gene in a subset of cases where tumor material was available. Previously published data suggest a high rate of LOH with loss of the normal allele in the majority of BRCA1 and BRCA2 cases with strong family history at approximately 80 and 70 years, respectively [24]. However, we did not find loss of either *BRCA1* or *BRCA2* in the majority of tumors. The low frequency of LOH was consistent with the results from a previously published case (case 12) where we did not find LOH of either gene in either the breast or ovarian tumor [27]. Three other papers on TH showed LOH with loss of the normal allele [1, 28, 35]. One potential reason for the low frequency of LOH in this study could be that seven of the breast tumor samples were areas of ductal carcinoma *in* situ (DCIS) with micro-invasion rather than a region of the invasive breast tumor. However, we identified two tumors with LOH in these types of samples so this explanation is unlikely to be the major cause of the low rate of LOH.

The observed ages at diagnosis of BC in TH, SH1, and SH2, and the distributions of tumor characteristics may also reveal the interactions of *BRCA1* and *BRCA2* mutations, which may have implications for modeling the cancer susceptibility in TH. The observed age distributions rule out a multiplicative model for the interactions of *BRCA1* and *BRCA2* mutations on BC risk. Given the well-established BC risks for *BRCA1* and *BRCA2* mutations, a multiplicative model would imply very high cancer risk at young ages. However, the present study suggests that ages at BC diagnosis in TH are not significantly different from those in *BRCA1* mutation carriers. Therefore, a multiplicative model of cancer risk for *BRCA1* and *BRCA2* is inconsistent with the current observations. This observation, combined with the fact that the tumor characteristics are intermediate to SH1 and SH2, suggests that an additive model for the joint effects of *BRCA1* and *BRCA2* mutations is more plausible. These results could be used for modeling the cancer risks for TH carriers and could be incorporated into risk prediction models.

Micro-satellite analysis alone did show a decrease in one of the two alleles in more of the tumors (6 out of 12 *BRCA1* and 5 out of 12 *BRCA2*); however, the sequencing data suggested that the mutant allele rather than the normal allele was lost in many of the tumors. Although the early publications in high-risk families showed very high rates of LOH, exclusively with loss of the normal allele, more recently there have been many publications showing larger numbers of cases with no LOH [19, 23, 24] and an increasing number of tumors with loss of the mutant allele [19, 24]. The second hit in these tumors could be due to a somatic mutation of the normal chromosome or due to promoter methylation, rather than LOH. Unfortunately, the amount of material from these tumors was very limited, and it was not possible to preform additional experiments to investigate alternative mechanisms. Methylation of *BRCA1* has been shown to be a mechanism of decreased *BRCA1* expression in sporadic BC [2, 34], although this is less frequent in *BRCA1* carriers [10, 33]. Why the mechanism of LOH with loss of the normal allele in TH might be different compared with SH is unclear. Tumor material was only available in a small proportion of the cases with cancer. Therefore, it is difficult to interpret the results of the tumor study more broadly. Despite the small numbers, we did not find evidence to support the hypothesis that the tumors would either have LOH of *BRCA1* or *BRCA2*. The TH breast tumor characteristics, however, do appear to be intermediate in phenotype to SH1 and SH2, suggesting some cancers are being driven by inactivation of *BRCA1* and some by inactivation of *BRCA2*. Additional studies that explore other causes of inactivation (e.g., methylation or somatic mutation) are warranted.

## Conclusions

We report evidence that the *BRCA1* mutation in TH may drive these clinical TH phenotypes based on elevated OC risk in TH vs. SH2 but not SH1, and earlier age of BC diagnosis in TH vs. SH2 but not SH1. Therefore, TH may be managed more like BRCA1 mutation carriers than BRCA2 mutation carriers. In contrast, TH breast tumor characteristics (e.g., ER/PR status) are intermediate in phenotype to SH1 and SH2. Future studies are warranted to understand whether TH should be managed differently to SH1 or SH2 carriers, and, if so, to enable individualized counseling and clinical management appropriate for TH mutation carriers.

## Additional files

Additional file 1: Table S1. Ethics committees that granted approval for the access and use of the data for this study. **Table S2.** Participant counts by center and mutation. **Table S3.** Primers used for PCR and Sanger sequencing. **Table S4.** Primers used in micro-satellite analysis for loss of heterozygosity. **Table S5.** Micro-satellite loss of heterozygosity and sequencing analysis results. (DOC 177 kb)

## Abbreviations

BC: Breast cancer
CIMBA: Consortium of Investigators of Modifiers of *BRCA1/2*
DCIS: Ductal carcinoma *in situ*
ER: Estrogen receptor
LOH: Loss of heterozygosity
OC: Ovarian cancer
PTC: Premature termination codon
PR: Progesterone receptor
RRSO: Risk-reducing salpingo-oophorectomy
SH1: Single mutation at *BRCA1*
SH2: Single mutation at *BRCA2*
TH: Transheterozygosity, transheterozygote
