# Supplementary material for: Inheritance of deleterious mutations at both BRCA1 and BRCA2 in an international sample of 32,295 women
Source: Breast Cancer Res. 2016 Nov 11;18:112. doi: 10.1186/s13058-016-0768-3 (PMC5106833; doi:10.1186/s13058-016-0768-3)
Supplement: Additional file 1: Table S1. — Ethics committees that granted approval for the access and use of the data for this study. Table S2. Participant counts by center and mutation. Table S3. Primers used for PCR and Sanger sequencing. Table S4. Primers used in micro-satellite analysis for loss of heterozygosity. Table S5. Micro-satellite loss of heterozygosity and sequencing analysis results. (DOC 177 kb) [file 13058_2016_768_MOESM1_ESM.doc]

**Table S1** Ethics committees that granted approval for the access and use of the data for this study

| **Study** | **Country** | **Committee approval** |
| --- | --- | --- |
| Breast Cancer Family Registry (BCFR) | USA | Institutional Review Board University of Utah |
| (BCFR - addtional) | Australia | The University of Melbnourne Health Sciences Human Ethics Sub-Committee |
| (BCFR - addtional) | USA | Columbia University Medical Center Institutional Review Board |
| (BCFR - addtional) | USA | Northern Californa Cancer Center Institutional Review Board |
| (BCFR - addtional) | Canada | University Health Network Research Ethics Board |
| (BCFR - addtional) | Canada | Mount Sinai Hospital Research Ethics Board |
| Baltic Familial Breast and Ovarian Cancer Consortium (BFBOCC) | Latvia,Lithuania | Centrālā medicīnas ētikas Komiteja |
| BRCA-gene mutations and breast cancer in South African women (BMBSA) | South Africa | Univ. of Pretoria and Pretoria Academic Hospitals Ethics Committee |
| Beckman Research Institute of the City of Hope (BRICOH) | USA | UC Irvine: Office of Research Administration Institutional Review Board |
| Copenhagen Breast Cancer Study (CBCS) | Denmark | De Videnskabsetiske Komiteer I Region Hovedsladen |
| Spanish National Cancer Centre (CNIO) | Spain | Instituto de Salud Carlos III Comité de Bioética y Bienestar Animal |
| CONsorzio Studi ITaliani sui Tumori Ereditari Alla Mammella (CONSIT TEAM) | Italy | Comitato Etico Indipendente della Fondazione IRCCS "Istituto Nazionale dei Tumori" |
| Deutsches Krebsforschungszentrum (DKFZ) | Germany | Ethik-Kommission des Klinikums der Universität |
| (DKFZ - addtional) | Columbia | Hospital Universitario de San Ignacio Comité de Investigaciones y Etica |
| (DKFZ - addtional) | Pakistan | Shaukat Khanum Memorial Cancer Hospital and Research Centre Institutional Review Board |
| HEreditary Breast and Ovarian study Netherlands (HEBON) | The Netherlands | Protocol Toetsingscommissie van het Nederlands Kanker Instituut/Antoni van Leeuwenhoek Ziekenhuis |
| Epidemiological study of BRCA1 and BRCA2 mutation carriers (EMBRACE) | UK and EIRE | Anglia & Oxford MREC |
| Fox Chase Cancer Center (FCCC) | USA | Institutional Review Board Fox Chase Cancer Center |
| German Consortium of Hereditary Breast and Ovarian Cancer (GC-HBOC) | Germany | Ethik-Kommission der Medizinischen Fakultät der Universät zu Köln |
| Georgetown University (GEORGETOWN) | USA | MedStar Research Institute - Georgetown University Oncology Institutional Review Board |
| Genetic Modifiers of cancer risk in *BRCA1*/2 mutation carriers (GEMO) | France | Comité consultatif sur le traitement de I'information en matière de recherche dans le domaine de la santé |
| Gynecologic Oncology Group (GOG) | USA | National Cancer Institute - Cancer Prevention and Control Concept Review Committee |
| Hospital Clinico San Carlos (HCSC) | Spain | Comité Ético de Investigación Clínia Hospital Clínico San Carlos |
| Helsinki Breast Cancer Study (HEBCS) | Finland | Helsingin ja uudenmaan sairaanhoitopiiri (Helsinki University Central Hospital ethics committee) |
| Hungarian Breast and Ovarian Cancer Study (HUNBOCS) | Hungary | Institutional Review Board of the Hungarian National Institute of Oncology |
| Univeristy Hospital Vall d'Hebron (HVH) | Spain | The Hospital Universitario Vall d'Hebron Clinical Research Ethics Committee |
| Institut Català d'Oncologia (ICO) | Spain | Catalan Institute of Oncology Institutional Review Board |
| Iceland Landspitali - University Hospital (ILUH) | Iceland | Vísindasiđanefnd National Boethics Committee |
| Interdisciplinary Health Research International Team Breast Cancer Susceptibility (INHERIT) | Quebec -Canada | Comité d'éthique de la recherche du Centre Hospitalier Universitaire de Québec |
| Istituto Oncologico Veneto Hereditary Breast and Ovarian Cancer Study (IOVHBOCS) | Italy | Centro Oncologico Regionale Azienda Ospedale Di Padova Comitato Etico |
| Kathleen Cuningham Foundation Consortium for Research into Familial Breast Cancer (KCONFAB) | Australia | Peter MacCallum Cancer Centre Ethics Committee |
| (KCONFAB - additional) | Australia | Queensland Institute of Medical Research - Human Research Ethics Committee |
| Modifiers and Genetics in Cancer (MAGIC) | USA | University of Pennsylvania Institutional Review Board |
| Mayo Clinic (MAYO) | USA | Mayo Clinic Institutional Review Boards |
| McGill University (MCGILL) | Canada | McGill Faculty of Medicine Institutional Review Board |
| Memorial Sloane Kettering Cancer Center (MSKCC) | USA | Memorial Sloan-Kettering Cancer Center IRB |
| (MSKCC - additional) | USA | Human Biospecimen Utilization Committee |
| Modifier Study of Quantitative Effects on Disease (MOD-SQUAD) | USA | Mayo Clinic Institutional Review Boards |
| General Hospital Vienna (MUV) | Austria | Ethikkommission der Medizinischen Universität Wien |
| National Cancer Institute (NCI) | USA | NIH Ethics Office |
| National Israeli Cancer Control Center (NICCC) | Israel | Carmel Medical Center Institutional Review Board (Helsinki Committee) |
| N.N. Petrov Institute of Oncology (NNPIO) | Russia | N.N. Petrov Institional Ethical Committee |
| Ontario Cancer Genetics Network (OCGN) | Canada | Mount Sinai Hospital Research Ethics Board |
| The Ohio State University Comprehensive Cancer Centre (OSU-CCG) | USA | Cancer Institutional Review Board |
| Odense University Hospital (OUH) | Denmark | Den Videnskabsetiske Komité for Region Syddanmark |
| Pisa Breast Cancer Study (PBCS) | Italy | Comitato Etico per lo studio del farmaco sull'uomo |
| Swedish Breast Cancer Study (SWE-BRCA) | Sweden | Regionala Etikprövningsnämnden Stockholm |
| University of California Irvine (UCI) | USA | UC Irvine: Office of Research Administration Institutional Review Board |
| University of California Los Angeles (UCLA) | USA | UCLA Institutional Review Board |
| University of California San Francisco (UCSF) | USA | Committee on Human Research |
| UK and Gilda Radner Familial Ovarian Cancer Registries (UKGRFOCR) | UK | Cambridge Local Research Ethics Committee |
| (UKGRFOCR - additional) | USA | Roswell Park Cancer Institute IRB |
| University of Pennsylvania (UPENN) | USA | University of Pennsylvania Institutional Review Board |
| Women’s Cancer Research Institute (WCRI) | USA | Cedars-Sinai Institutional Review Board |

**Table S2**: Participant Counts by Center and Mutation

| **Study** | **Single *BRCA1* or *BRCA2* Carrier** | **Dual *BRCA1+BRCA2* Carrier** | **Total** |
| --- | --- | --- | --- |
|  |  |  |  |
| BCFR-AU | 80 | 1 | 81 |
| BCFR-NC | 63 | 4 | 70 |
| BCFR-NY | 167 | 1 | 168 |
| BCFR-ON | 116 | 1 | 120 |
| BCFR-PA | 74 | 1 | 77 |
| BCFR-UT | 74 | 0 | 76 |
| BFBOCC | 236 | 0 | 236 |
| BIDMC | 75 | 1 | 76 |
| BMBSA | 40 | 0 | 40 |
| BRICOH | 100 | 2 | 103 |
| CBCS | 117 | 0 | 117 |
| CNIO | 191 | 0 | 193 |
| COH | 186 | 2 | 190 |
| CONSIT TEAM | 385 | 5 | 390 |
| DEMOKRITOS | 103 | 1 | 88 |
| DFCI | 171 | 0 | 175 |
| DKFZ | 145 | 0 | 145 |
| HEBON | 380 | 0 | 405 |
| EMBRACE | 776 | 14 | 795 |
| FCCC | 119 | 2 | 123 |
| GC-HBOC | 920 | 10 | 986 |
| GEMO | 657 | 2 | 670 |
| GEORGETOWN | 53 | 1 | 54 |
| NRG_ONCOLOGY | 315 | 1 | 326 |
| HCSC | 109 | 1 | 111 |
| HEBCS | 20 | 0 | 20 |
| HRBCP | 13 | 0 | 13 |
| HUNBOCS | 162 | 2 | 164 |
| HVH | 30 | 0 | 30 |
| ICO | 112 | 0 | 112 |
| IHCC | 1,503 | 0 | 1,503 |
| INHERIT | 110 | 0 | 110 |
| IOCHBOCS | 104 | 1 | 104 |
| IPOBCS | 20 | 0 | 20 |
| KCONFAB | 351 | 4 | 363 |
| KOHBRA | 65 | 4 | 71 |
| MAGIC | 102 | 1 | 103 |
| MAYO | 195 | 1 | 200 |
| MCGILL | 56 | 0 | 56 |
| UTMDACC | 61 | 0 | 61 |
| MODSQUAD | 308 | 0 | 308 |
| MSKCC | 462 | 2 | 465 |
| MUV | 277 | 5 | 289 |
| UPITT | 0 | 2 | 2 |
| NCI | 107 | 0 | 108 |
| NNPIO | 129 | 0 | 129 |
| OCGN | 160 | 0 | 168 |
| OSU CCG | 72 | 1 | 76 |
| OUH | 111 | 1 | 112 |
| PBCS | 22 | 0 | 22 |
| SEABASS | 15 | 0 | 15 |
| SMC | 1,173 | 8 | 1,181 |
| SWE-BRCA | 276 | 2 | 278 |
| UCHICAGO | 62 | 0 | 63 |
| UCLA | 91 | 0 | 92 |
| UCSF | 115 | 0 | 116 |
| UKGRFOCR | 65 | 0 | 65 |
| UPENN | 349 | 4 | 354 |
| VFCTG | 74 | 0 | 74 |
| WCP | 262 | 5 | 267 |
| Total | 12,686 | 93 | 12,929 |
|  |  |  |  |

**Table S3**: Primers used for PCR and Sanger sequencing

| **Gene** | **HGVS:  genomic level** | **BIC "style": genomic level** | **Primer F 5’-3’** | **Primer R 5’-3’** | **Length bp** | **Annealing Temperature °C** |
| --- | --- | --- | --- | --- | --- | --- |
| *BRCA1* | c.5136G>A | 5255G>A (W1712X) | TGCAATTCTGAGGTGTTAAAGGGA | GGACAGCAcTTCCTGATTTTGTT | 190 | 60 |
| *BRCA1* | c.68_69delAG | 185delAG-ter39 | TGTCTTTTCTTCCCTAGTATGT | ATGTGTTAAAGTTCATTGGAACAGAA | 209 | 57 |
| *BRCA1* | c.181T>G | 300T>G-Cys61Gly | TTTCCTACTGTGGTTGCTTCCAA | TCATGGCTATTTGCCTTTTGAG | 208 | 57 |
| *BRCA1* | c.5251C>T | 5370C>T (R1751X) | TCAACTTGAGGGAGGGAGCTTTA | ATATGACGTGTCTGCTCCACTTC | 188 | 60 |
| *BRCA1* | c.5266dupC | 5382insC-ter1829 | TCAACTTGAGGGAGGGAGCTTTA | ATATGACGTGTCTGCTCCACTTC | 188 | 60 |
| *BRCA1* | c.3700_3704del5 | 3819del5-ter1241 | TCAATGATAATAAATTCTCCTCTGTGTTCT | AGTGAGGATGAAGAGCTTCCC | 141 | 57 |
| *BRCA1* | c.1793T>A | 1912T>A (L598X) | TTTTAGGTGCTTTTGAATTGTGGA | CGGAGCAGAATGGTCAAGTGAT | 210 | 57 |
| *BRCA2* | c.8537_8538delAG | 8765delAG-ter2867 | TGTGACTTTTTTGGTGTGTGTAA | ACCTTcATGTTCTTCAaATTCCTCCT | 184 | 57 |
| *BRCA2* | c.4965delC | 5193delC | TGAAAGTTAAAGTACATGAAAATGTAGAAAAA | GGTTGACCATCAAATATTCCTTCTC | 203 | 57 |
| *BRCA2* | c.5946delT | 6174delT-ter2003 | TCAGTCTCATCTGCAAATACTTGTG | TGTGAGCTGGTCTGAATGTT | 180 | 57 |
| *BRCA2* | c.1318_1319dupCT | 1546dupCT | AATCTCCAAGGAAGTTGTACCG | GGCTAGAAaTAcGTGGCAAAGAA | 210 | 60 |
| *BRCA2* | c.6753_6754delTT | 6981delTT | CTTTGAAACAGAAGCAGtAGAAATTG | GGCAACACGAAAGGTAAAAATGAAC | 208 | 60 |
| *BRCA2* | c.8363G>A | 8591G>A | CTTTTTAAAGTGAATATTTTTAAGGCAGTTCTA | AGGAAAAGGTCtaGGGTCAGGAA | 192 | 60 |
| *BRCA2* | c.681+1G>A | IVS8+1G>A | TGTGTCATGTAATCAAATAGTAGATGTG | AGCAATTTCAACAGTCTAATCAATGTC | 194 | 57 |

**Table S4**: Primers used in micro-satellite analysis for loss of heterozygosity

| **Name** | **Gene** | **Primer-F 5’-3’** | **Primer-R 5’-3’** | **Label** | **Distance**  ***BRCA1* or *BRCA2*** | **Allele size range bp** | **PCR temp** | **Hetero-zygosity** |
| --- | --- | --- | --- | --- | --- | --- | --- | --- |
| D17S1322 | *BRCA1* | CTAGCCTGGGCAACAAACGA | GCAGGAAGCAGGAATGGAAC | VIC | intron 19 of *BRCA1* | 108-125 | 57 | 0.66 |
| D17S855 | *BRCA1* | GGATGGCCTTTTAGAAAGTGG | ACACAGACTTGTCCTACTGCC | NED | intron 20 of *BRCA1* | 138-157 | 57 | 0.82 |
| D13S290 | *BRCA2* | CCTTAGGCCCCATAATCT | CAAATTCCTCAATTGCAAAAT | FAM | 1.46 MB centromeric of *BRCA2* | 171-185 | 57 | 0.46 |
| D13S260 | *BRCA2* | TCAGATTGCTAAGCATGTACC | CATTTAGAGTTATACGTCTCCCAGA | FAM | 0.45 MB centromeric of *BRCA2* | 158-173 | 52 | 0.78 |
| D13S1698 | *BRCA2* | GTCCATACCACTAAGTCTGAC | AACCTCAGGCTAATAGTCTCA | FAM | 0.18 MB centromeric of *BRCA2* | 123-140 | 57 | 0.63 |
| D13S171 | *BRCA2* | CCTACCATTGACACTCTCAG | TAGGGCCATCCATTCT | FAM | 0.28 MB telomeric of *BRCA2* | 227-241 | 57 | 0.72 |

The **h**eterozygosity for these markers in *BRCA1* and *BRCA2* from the Genome Database no longer available were published previously(Khoo, et al., 2002; Miolo, et al., 2006). The distance from *BRCA1/2* is also published in these references.

**Table S5: Micro-satellite loss of heterozygosity and sequencing analysis results**

| **Case** | **D17S**  **1322**  ***BRCA1*** | **D17S**  **855**  ***BRCA1*** | **Micro**  **LOH *BRCA1*** | **Sequence**  ***BRCA1*** | **D13S**  **290**  ***BRCA2*** | **D13S**  **260**  ***BRCA2*** | **D13S**  **1698 *BRCA2*** | **D13S**  **171**  ***BRCA2*** | **Micro LOH *BRCA2*** | **Sequence**  ***BRCA2*** |
| --- | --- | --- | --- | --- | --- | --- | --- | --- | --- | --- |
| 3 | **0.46** | NI | **Yes** | mut < N | NI | 0.79 | NI | NI | No | equal |
| 5 | 1.02 | 0.83 | No | equal | NI | 0.94 | 1.04 | 0.64 | No | **N < mut** |
| 6 L | NI | 1.11 | No | equal | NI | 1.34 | 0.77 | NI | No | equal |
| 6 R | NI | **0.49** | **Yes** | mut < N | NI | 1.04 | 1.07 | NI | No | equal |
| 7 | 1.53 | 0.66 | No | **N < mut** | 1.56 | 1.29 | NI | NI | No | mut < N |
| 8 | **0.52** | **0.52** | **Yes** | **N < mut** | **0.27** | NA | NA | fail | **Yes** | equal |
| 9 | 0.69 | 0.67 | No | **N < mut** | NI | 0.72 | **1.73** | NI | **Yes** | **N < mut** |
| 10 | NI | **2.14** | **Yes** | fail | **5.99** | NI | NI | NI | **Yes** | fail |
| 1 | **0.37** | **2.29** | **Yes** | Only N | **2.05** | fail | fail | fail | **Yes** | equal |
| 2 ov | NI | **14.24** | **Yes** | **Only mut** | **4.03** | NI | 1.23 | fail | No | Only N |
| 2 br | NI | 1.05 | No | **N < mut** | **9.37** | NI | **11.98** | **4.72** | **Yes** | mut < N |
| 4 | 1.52 | 0.64 | No | equal | 0.89 | 0.65 | 1.34 | NI | No | equal |

For cases, in the bilateral breast cancer case L is left breast tumor and R is right breast tumor. In the case with breast and ovarian cancer ov is the ovarian tumor and br is the breast tumor. The microsatellite LOH score at each marker is indicated in bold if LOH is present indicated by L <0.6 or L > 1.67. Micro LOH for each gene is a combination of all markers in the region with yes indicated in bold. Sequencing analysis for each gene indicates if the two alleles in the tumor at the mutation position are equal peak heights compared to the germline sample and if not which allele was lower. N is the normal allele and mut is the mutant allele. Bold indicates the normal allele is decreased or lost. Grey shading indicated that there is LOH by microsatellite analysis and the sequencing analysis shows loss or decrease of the normal allele.
